# The Clinical Value of Poor-Law Infirmaries

**Published:** 1915-02-27

**Authors:** 


### The Clinical Value of Poor-Law Infirmaries.

Note.—On this subject further communications have been received, but as no new points are made, the correspondence must close. We hope to publish a leading article on the controversy next week.—Ed. The Hospital.

